# Platelet-Derived Mitochondria Attenuate 5-FU-Induced Injury to Bone-Associated Mesenchymal Stem Cells

**DOI:** 10.1155/2023/7482546

**Published:** 2023-01-30

**Authors:** Yutong Chen, Zhixin Liu, Ning An, Jing Zhang, Weicheng Meng, Wenting Wang, Xiaoshuang Wu, Xingbin Hu, Yaozhen Chen, Wen Yin

**Affiliations:** Department of Transfusion Medicine, Xijing Hospital, Fourth Military Medical University, Xi'an 710032, China

## Abstract

**Background:**

Myelosuppression is a common condition during chemotherapy. Bone-associated mesenchymal stem cells (BA-MSCs) play an essential role in the composition of the hematopoietic microenvironment and support hematopoietic activity. However, chemotherapy-induced damage to BA-MSCs is rarely studied. Recent studies have shown that platelets promote the wound-healing capability of MSCs by mitochondrial transfer. Therefore, this study is aimed at investigating the chemotherapy-induced damage to BA-MSCs and the therapeutic effect of platelet-derived mitochondria. *Material/Methods*. We established *in vivo* and *in vitro* BA-MSC chemotherapy injury models using the chemotherapy agent 5-fluorouracil (5-FU). Changes in the mitochondrial dynamics were detected by transmission electron microscopy, and the expression of mitochondrial fusion and fission genes was analyzed by qRT-PCR. In addition, mitochondrial functions were also explored by flow cytometry and luminometer. Platelet-derived mitochondria were incubated with 5-FU-damaged BA-MSCs to repair the injury, and BA-MSC functional changes were examined to assess the therapy efficacy. The mechanism of treatment was explored by studying the expression of mitochondrial fission and fusion genes and hematopoietic regulatory factor genes in BA-MSCs.

**Results:**

Stimulation with 5-FU increased the apoptosis and suppressed cell cycle progression of BA-MSCs both *in vivo* and *in vitro*. In addition, 5-FU chemotherapy inhibited the hematopoietic regulatory ability and disrupted the mitochondrial dynamics and functions of BA-MSCs. The mitochondrial membrane potential and ATP content of 5-FU-injured BA-MSCs were decreased. Interestingly, when platelet-derived mitochondria were transferred to BA-MSCs, the 5-FU-induced apoptosis was alleviated, and the hematopoietic regulatory ability of 5-FU-injured BA-MSCs was effectively improved by upregulating the expression of mitochondrial fusion genes and hematopoietic regulatory factor genes.

**Conclusion:**

BA-MSCs were severely damaged by 5-FU chemotherapy both *in vivo* and *in vitro*. Meanwhile, platelet-derived mitochondria could attenuate the 5-FU-induced injury to BA-MSCs, which provides future research directions for exploring the treatment strategies for chemotherapy-injured BA-MSCs and establishes a research basis for related fields.

## 1. Introduction

Chemotherapy is a common treatment for hematological neoplasms, especially before bone marrow transplantation [1–3]. Chemotherapy not only eliminates tumor cells to achieve the goal of tumor treatment but is also used to eliminate the patient's own hematopoietic stem cells (HSCs) to reduce the risk of graft-vs-host-disease (GVHD) after transplantation [4–6]. Myelosuppression often occurs after chemotherapy, and the main reason is chemotherapy-induced injury of bone marrow sinusoidal endothelium [7]. In other words, chemotherapy damages the hematopoietic microenvironment, which negatively affects bone marrow homeostasis and bone marrow hematopoietic function [8–10].

Studies have shown that in addition to killing tumor cells, chemotherapy drugs damage the bone marrow hematopoietic microenvironment [9, 11]. In 1978, Schofield first proposed the concept of the bone marrow hematopoietic niche [12]. He found that the niche was involved in the maintenance, self-renewal, and regulation of multidifferentiation of HSCs [13]. Bone marrow mesenchymal stem/stromal cells (MSCs), the main component of the niche, are multipotent cells characterized by self-renewal and multilineage differentiation potential [14]. MSCs also provide hematopoietic support [15], promote stem cell implantation [16, 17], contribute to immune regulation [18, 19], and perform other functions. The chemotherapy-caused injury of MSCs affects immune reconstitution and hematopoietic function recovery of donor HSCs in the host hematopoietic microenvironment after HSC transplantation [20]. Bone-associated mesenchymal stem cells (BA-MSCs), which are derived from the endosteum, are an essential component for the formation and maintenance of the hematopoietic microenvironment and play an important role in the regulation and support of hematopoietic activity [21, 22]. Therefore, an in-depth study of the mechanism of BA-MSC injury caused by chemotherapy is crucial to improve the success rate of clinical HSC transplantation, promote immune reconstitution, and reduce the risk of postoperative infection.

Currently, the use of MSCs has become the main strategy in stem cell therapy [23]. In the clinical application, when stimulated by the injury signals released by damaged cells, MSCs can transfer mitochondria to injured cardiomyocytes, neuronal cells, alveolar epithelial cells, and other cells associated with specific tissue and organ diseases [24, 25]. The transfer of MSC-derived mitochondria improves the functional activity of mitochondria in injured cells to help produce more adenosine triphosphate (ATP), reduce the production of reactive oxygen species, and promote the self-healing of cells [24, 26, 27]. However, stem cell therapy is not always satisfactory owing to a low cell survival and poor engraftment. Stem cells for treatment must be appropriately adapted to the harsh *in vivo* environment [28]. The transfer of exogenous mitochondria to adipose-derived mesenchymal stem cells (ADSCs) can effectively improve ADSC energy levels, thus achieving effective cell implantation [29]. Therefore, we hypothesized that exogenous mitochondria could be transferred to BA-MSCs damaged by chemotherapy drug 5-fluorouracil (5-FU) to repair chemotherapy-induced cell damage. This innovation may be an effective method to repair the chemotherapy damage of BA-MSCs.

In addition to the physiological role of platelets in hemostasis [30], previous studies have found that platelet derivatives can be used as fetal bovine serum (FBS) substitute for MSCs cultured and expanded *in vitro* [31] and have the characteristics of improving the wound repair ability of MSCs [32]. There are more functional characteristics of platelets worth to be explored, and it has a broader application prospect. Levoux et al. reported a novel mechanism by which platelets enhance the regenerative capacity of MSCs. They found that activated platelets released respiratory functional mitochondria into MSCs, improving the proangiogenic function of MSCs by increasing cytosolic citric acid levels and stimulating fatty acid synthesis. This process enhanced the therapeutic efficacy of MSCs in skin wound healing in mice [33]. Previous studies have shown that mitochondria in MSCs can repair cell damage through intercellular mitochondrial transfer [24]. Levoux et al. demonstrated that platelet mitochondria can be successfully transferred to MSCs and show favorable metabolic regulation ability and promote wound repair ability of MSCs. However, it is still unclear whether platelet mitochondria can enhance hematopoietic regulatory properties of MSCs and whether it is feasible to apply platelet-derived mitochondria in the treatment of chemotherapy-injured BA-MSCs. We believe that by studying the transfer of platelet-derived mitochondria to BA-MSCs for the treatment of chemotherapy-damaged BA-MSCs, the barrier between cells can be further broken, and the application potential of platelets can be further broadened. It is of great significance to demonstrate the repair effect of platelet-derived mitochondria on chemotherapy injury of BA-MSCs and explore the mechanism of platelet-derived mitochondria in the treatment of chemotherapy injury of BA-MSCs.

BA-MSCs are a long-term research object of our research group. In the present study, we demonstrated that 5-FU chemotherapy induced the apoptosis, inhibited cell cycle progression, and impaired the hematopoietic regulatory function of BA-MSCs. In addition, the mitochondrial dynamics and functions of BA-MSCs were damaged. The transfer of platelet-derived mitochondria to 5-FU-injured BA-MSCs could alleviate the apoptosis and improve the hematopoietic regulatory ability of BA-MSCs. Our findings highlight the severity of chemotherapy-induced BA-MSC injury and the necessity to repair BA-MSC injury during chemotherapy. Simultaneously, the effectiveness and importance of mitochondrial transplantation in the treatment of chemotherapy-injured BA-MSCs were confirmed. In the future, the specific mechanism of mitochondrial treatment remains to be explored.

## 2. Materials and Methods

### 2.1. Mice

Female C57BL/6 mice, aged 8-12 weeks, were obtained from the Laboratory Animal Center of Fourth Military Medical University (Xi'an, China). Mice were fed in an environment free of specific pathogens and managed according to standards [34]. The experiments performed on mice were reviewed and approved by the Animal Care and Use Committee of Fourth Military Medical University. To establish a myelosuppression mice model, mice were intraperitoneally injected with 5-FU (150 mg/kg) [35, 36] dissolved in 200 *μ*L phosphate buffer saline (PBS) as a 5-FU injury group; control group mice were intraperitoneally injected with 200 *μ*L PBS per mouse. The injury of bone marrow cells (BMCs) and BA-MSCs was detected after 6 days. For *in vivo* studies, mice were randomly assigned to each group. Investigators were blinded to mice allocation during the experiments as well as during the analysis.

### 2.2. Reagents

The apoptosis levels of BA-MSCs and BMCs were detected with FITC Annexin V Apoptosis Detection Kit (#556547, BD Biosciences). The Cell Cycle and Apoptosis Analysis Kit (#C1052, Beyotime) with propidium (PI) staining was used to analyze the cell cycle of BA-MSCs and BMCs. The anti-mouse antibodies used for flow cytometry analysis were as follows: CD11b-APC (#101212, Biolegend), Gr-1-APC (#108412, Biolegend), CD3-APC (#100236, Biolegend), CD19-APC (#17-0193-80, eBioscience), Ter119-APC (#17-5921, eBioscience), B220-APC (#103212, Biolegend), Sca-1-PE-Cy7 (#558162, BD Biosciences), c-Kit-PE (#105807, Biolegend), CD16/32-Percp-Cy5.5 (#560540, BD Biosciences), CD34-FITC (#343504, Biolegend), and Flk2-BV421 (#135313, Biolegend). The anti-mouse antibodies used for immunoblotting assays were as follows: OPA1 (#80471, Cell Signaling Technology), DRP1 (#EPR19274, Abcam), TOMM20 (#EPR15581, Abcam), COX IV (#38563, Cell Signaling Technology), alpha tubulin (#66031-1-1 g, Proteintech), GAPDH (#97166, Cell Signaling Technology), HRP goat anti-mouse IgG antibody (#EK010, Zhuangzhibio), and HRP goat anti-rabbit IgG antibody (#EK020, Zhuangzhibio). Mitochondrial Membrane Potential Assay Kit with JC-1 (#C2006, Beyotime) was used to determine mitochondrial membrane potential (MMP) of BA-MSCs and platelet-derived mitochondria. ATP Assay Kit (#S0026, Beyotime) was used to analyze the ATP level of BA-MSCs.

### 2.3. BA-MSC Isolation and Culture

Mice aged 8-12 weeks were used to isolate BA-MSCs and BMCs. To obtain BA-MSCs and BMCs, the tibia and femur of mice were extracted, and then the muscle and connective tissue were removed. The two ends of the bone were cut off, and the bone marrow was gently flushed out with a 1 mL sterile syringe containing PBS. The remaining bone was digested with collagenase I (#9001-12-1, Sigma) on a shaker at 37°C and 180 RPM for 1 h to obtain BA-MSCs. BA-MSCs were cultured in MEM-alpha medium (Gibco) containing 15% FBS (Gibco) in an incubator with 5% CO_2_ at 37°C. The isolated BA-MSCs were identified as previously reported [21]. The red blood cells (RBC) in the BMCs were lysed with ammonium-chloride-potassium (ACK) lysis buffer (#C3702, Beyotime), and then the BMCs were cultured in IMDM medium (Gibco) containing 15% FBS in an incubator with 5% CO_2_ at 37°C. BA-MSCs were stimulated with 12.5 *μ*M 5-FU for 48 h to establish the cell model of BA-MSCs injured by chemotherapy *in vitro* [37, 38]. BA-MSCs with no more than 5 passages were used in all experiments. The control group BA-MSCs were stimulated with dimethyl sulfoxide (DMSO) (#196055, MP Biomedicals). BA-MSCs (1 × 10^5^ cells/well) in MEM-alpha medium were seeded into a 12-well plate. After overnight culture for adhesion of BA-MSCs, BMCs were added to the supernatant at a ratio of 10 : 1 (1 × 10^6^ cells/well). After 2 and 5 days of coculture, the cells in the supernatant were taken for analysis.

OP9 cell line (ATCC CRL-2749) was cultured in our laboratory. Cells were incubated in MEM-alpha medium (Gibco) containing 10% FBS at 37°C in an atmosphere of 5% CO_2_. OP9 cells were stimulated with 12.5 *μ*M, 25 *μ*M, and 50 *μ*M 5-FU for 24 h and 48 h, respectively. The control group was supplemented with the same amount of DMSO.

### 2.4. Morphological Analysis

The 5-FU-injured BA-MSCs and the control BA-MSCs were digested in 25 cm^2^ culture flasks, washed once with PBS, and fixed with 2.5% glutaraldehyde. The samples were sent to the State Key Laboratory of Military Stomatology of Fourth Military Medical University and examined using a transmission electron microscope (TEM) (Tecnai G2, FEI Company, USA).

### 2.5. HSPCs' Primitive Population Flow Cytometry Analysis

To analyze the hematopoietic stem/progenitor cells' (HSPCs') primitive population of BMCs, BMCs were isolated from mice and then lysed with ACK lysis buffer to eliminate RBCs or obtained from *in vitro* coculture systems. The harvested BMCs were then resuspended in PBS supplemented with 5% FBS and 0.4% NaN3. To assess the lineage cell populations of BMCs, specific antibodies against CD3, CD11b (Mac-1*α*), CD19, B220 (CD45R), Gr-1 (Ly-6G/C), and Ter119 were used. To evaluate the HSPCs' primitive population, cells were stained with antibodies c-Kit- (CD117) PE, Sca-1- (Ly 6A/E) PE-Cy7, CD34-FITC, CD16/32-Percp-Cy5.5, and Flk2- (CD135) BV421. And then, the following procedure was performed: cells were stained with surface marker specific antibodies for 20 min at 4°C in darkness, and the residual antibodies were removed by washing three times with PBS. The stained cells were detected and analyzed with BD FACSCanto Plus flow cytometer (BD Biosystems, USA), and the data were collected and processed with FlowJo software (version 10).

### 2.6. Quantitative Real-Time PCR

RNAs were extracted from BA-MSCs by using the RNeasy RNA Isolation Mini Kit (#74104, Qiagen). Reverse transcription was performed using the Sensiscript RT kit (#205211, Qiagen) according to the manufacturer's instructions. Quantitative real-time PCR (qRT-PCR) reactions were performed with the Tianlong Real-Time PCR System by using the Fast Essential DNA Green Master Kit (#06402712001, Roche). The qRT-PCR conditions were as follows: at 95°C for 10 min; and then 40 cycles, including one step at 95°C for 20 seconds, one step at 55°C for 20 seconds, and 72°C for 20 seconds; and the last cycle at 95°C for 10 seconds, 65°C for 1 min, and 97°C for 1 second. Relative expression of genes was determined by cycle threshold (Ct) value and normalized to the internal control GAPDH. The primer sequences used in this study are listed in Table S1.

### 2.7. Western Blotting

Cells were lysed with RIPA lysis buffer (#P0013C, Beyotime) at 4°C for 30 min. Protein concentrations were determined using the BCA Protein Assay Kit (#P0012S, Beyotime). After that, the proteins were denatured using loading buffer (#P0015L, Beyotime) by heating at 100°C for 10 min. Protein samples of equal protein concentration were separated by SDS-PAGE and then transferred to PVDF membrane. PVDF membranes were blocked with PBST blocking buffer containing 5% skim milk for 2 h at room temperature, then incubated with specific primary antibodies (as described above) overnight at 4°C. After incubated with appropriate HRP-conjugated secondary antibodies for 1 h at room temperature, bands were imaged using an ECL kit (#34077, Thermo Scientific).

### 2.8. Platelet Preparation and Mitochondrial Isolation

Human apheresis platelets were obtained from Department of Blood Transfusion, Xijing Hospital. To obtain pure platelets and a sufficient quantity of mitochondria, 1 mL apheresis platelets were washed once with PBS containing 10% ACD (ACD solution contained 1.33 g sodium citrate, 0.47 g citrate, and 3 g glucose per 100 mL), and the plasma was removed by centrifugation at 1800 RPM for 5 min. The collected pellets were prepared for mitochondria isolation.

Platelet-derived mitochondria were isolated by using Qproteome Mitochondrial Isolation Kit (#37612, Qiagen). The steps of extracting mitochondria were as follows: step 1: platelet pellet was resuspended in 1 mL ice-cold lysis buffer and incubated with continuous shaking at 4°C for 10 min, followed by centrifugation of the lysate at 1000 g for 10 min at 4°C; step 2: carefully removed the supernatant, and then the platelet pellet was resuspended in 1 mL ice-cold disruption buffer and mixed thoroughly. Next, the platelets were completely destroyed using a blunt-ended needle and a syringe by slowly drawing the lysate into the syringe and then ejecting it with one stroke and repeated it for 10 times. Lysates were centrifuged at 1000 g for 10 min at 4°C, and the supernatant was carefully transferred to a clean 1.5 mL tube; step 3: the supernatant from step 2 was centrifuged at 6000 g for 10 min at 4°C and then remove the supernatant carefully. The mitochondrial pellet at the bottom of the tube was cleaned with 1 mL of mitochondrial storage buffer by carefully pipetting up and down using a 1 mL pipette tip. The supernatant was removed after centrifugation at 6000 g for 20 min at 4°C, and the mitochondrial pellet obtained was at the bottom of the tube. The isolated mitochondria were resuspended in mitochondrial storage buffer for the next experimental procedures. All procedures were conducted in accordance with the requirements of the Ethics Committee of Xijing Hospital.

### 2.9. Identification of Isolated Mitochondria

After mitochondria were isolated from platelets, the purity of extracted mitochondria was evaluated by western blotting assay to detect the contents of mitochondrial reference proteins TOMM20 and COX IV and cytoplasmic proteins GAPDH. The function of isolated mitochondria was evaluated by measuring MMP. The operation steps of MMP detection of isolated mitochondria were carried out according to the manufacturer's instructions and analyzed by Synergy LX Multimode Reader (BioTek, USA). Mitochondria and platelets were labeled with MitoTracker Red CMXRos (#C1049B, Beyotime), respectively, and then the size and morphology of isolated mitochondria and platelets were detected by using the Olympus FluoView™ FV1000 confocal laser scanning microscope (Olympus, Tokyo, Japan).

### 2.10. Mitochondria Transfer Evaluation

To evaluate the transfer of human platelet-derived mitochondria to mouse BA-MSCs, BA-MSCs were seeded in 12-well glass slides with 2 × 10^5^ cells per well. The cells were cultured overnight until all cells adhered to the wall. MitoTracker Red CMXRos labeled mitochondria were cocultured with BA-MSCs. At 4 h and 8 h, the transfer of mitochondria into BA-MSCs was detected using Pannoramic MIDI II scanner (3DHISTECH, Hungary). Before detection, the cytoplasm of BA-MSCs was labeled with carboxyfluorescein diacetate and succinimidyl ester (CFSE) (#C1031, Beyotime), and the nucleus of BA-MSCs was labeled with DAPI (#C1002, Beyotime).

### 2.11. ATP Assay

Intracellular ATP levels were evaluated using the ATP Assay Kit (#S0026, Beyotime) according to the manufacturer's instructions. The luminescence (RLU) value was detected by Synergy LX Multimode Reader (BioTek, USA). The concentration of ATP was calculated from the standard curve based on the RLU values. The number of cells used to analyze ATP concentration remained consistent between groups.

### 2.12. MMP Detection

Cells were digested from the 12-well plate with Trypsin-EDTA (#2360155, Gibco) and washed once with PBS. 10^5^ cells were resuspended in 0.5 mL MEM-alpha medium. And then, 0.5 mL JC-1 staining solution was added, thoroughly mixed, and incubated at 37°C for 20 min in a cell incubator. After incubation, the cells were precipitated by centrifugation at 600 g for 5 min at 4°C. Discard the supernatant and be careful not to lose cells. The cells were washed twice with JC-1 staining buffer (1×), resuspended with an appropriate amount of JC-1 staining buffer (1×), and analyzed by Beckman Coulter Epics XL flow cytometer (Beckman Coulter, USA).

### 2.13. Apoptosis and Cell Cycle Detection

For apoptosis analysis, cells were digested from the 12-well plate with Trypsin-EDTA (#2360155, Gibco) and washed twice with cold PBS. Resuspend cells with binding buffer (1×) and adjust the concentration to 10^6^ cells/mL. Take 100 *μ*L binding buffer (1×) (10^5^ cells) and place them in a new tube. Add 5 *μ*L annexin V and 5 *μ*L PI into the cells suspension and mix them evenly. After mixing, the cell suspension was incubated for 15 min at room temperature in the dark. At the end of the incubation, 400 *μ*L of binding buffer (1×) was added to the cell suspension, and the level of apoptosis was detected by BD FACSCanto Plus flow cytometer (BD Biosystems, USA) within 1 h.

For cell cycle detection, cells were digested from the 12-well plate and washed once with cold PBS. Cells were precipitated by centrifugation at 1000 g for 3 min and then fixed with precooled 70% ethanol at 4°C for 12 h. After 12 h, ethanol was removed by centrifugation, and the cells were washed once with cold PBS. The cells were incubated with PI staining solution at 37°C for 30 min, and then the cell cycle was detected by BD FACSCanto Plus flow cytometer (BD Biosystems, USA).

### 2.14. Statistical Analysis

Data are expressed as mean ± standard error of the mean (SEM). Data analysis was performed using GraphPad Prism software version 8.0 (San Diego, CA). For comparisons between two groups, data were analyzed by Student's *t*-test. For statistical analysis of multiple groups, one-way analysis of variance (one-way ANOVA) was applied. Results of statistical analysis are considered significant when *p* < 0.05. The statistical method used for each experiment is presented in each figure legend.

## 3. Results

### 3.1. 5-FU Induces BA-MSC Apoptosis *In Vitro* and *In Vivo*

**Figure 1:**
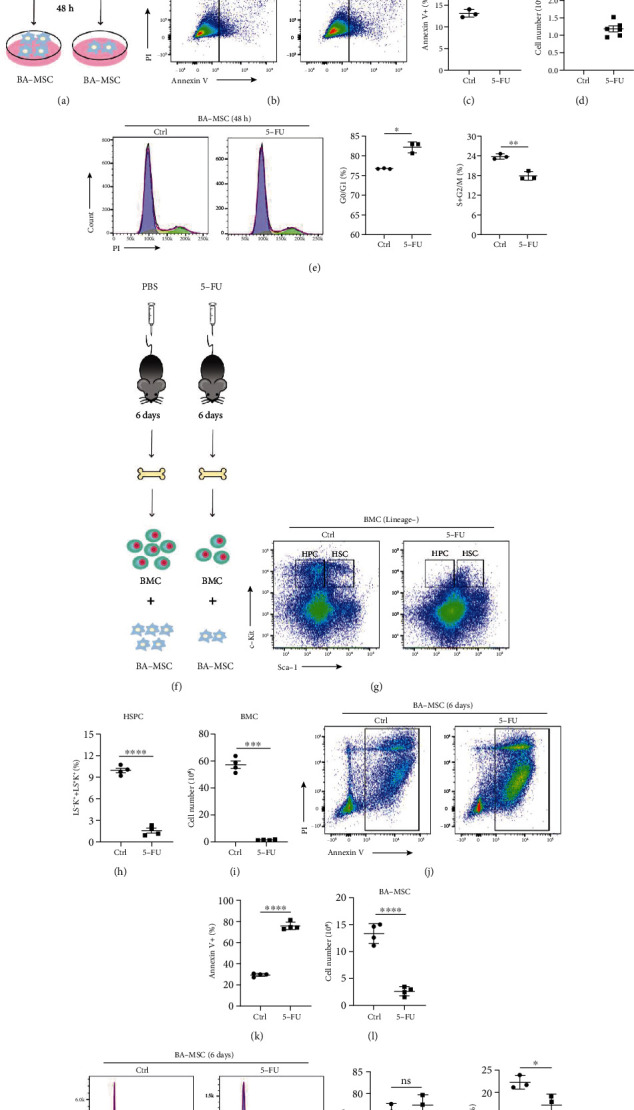
5-FU induces BA-MSCs injury *in vitro* and *in vivo*. (a) Schematic of BA-MSC injury model induced by 5-FU *in vitro*. (b) Flow cytometric plots of apoptosis detection of BA-MSCs injured by 5-FU. (c) Flow cytometry analysis of annexin V^+^ BA-MSC frequency in 5-FU-induced BA-MSC injury model *in vitro* (*n* = 3). (d) Analysis of total cell number of BA-MSCs in 5-FU-induced BA-MSC injury model *in vitro* (*n* = 6). (e) Flow cytometric plots of cell cycle and BA-MSC cell cycle statistical analysis in 5-FU-induced BA-MSC injury model *in vitro* (*n* = 3). (f) Schematic of mouse myelosuppression model induced by 5-FU *in vivo*. (g) Flow cytometric plots of BMC subsets (HSCs and HPCs). (h) Statistical analysis of percentage of HSPCs in BMCs in myelosuppression mouse model (*n* = 4). (i) Analysis of total cell number of BMCs in myelosuppression mouse model (*n* = 4). (j) Flow cytometric plots of apoptosis detection of BA-MSCs in myelosuppression mouse model. (k) Flow cytometry analysis of annexin V^+^ BA-MSC frequency in myelosuppression mouse model (*n* = 4). (l) Analysis of total cell number of BA-MSCs in myelosuppression mouse model (*n* = 4). (m) Flow cytometric plots of cell cycle and BA-MSC cell cycle statistical analysis in myelosuppression mouse model (*n* = 3). (a–e) BA-MSCs in 5-FU group were treated with 12.5 *μ*M 5-FU for 48 h; BA-MSCs in control group were treated with 12.5 *μ*M DMSO for 48 h. (f–m) Mice of 5-FU group were intraperitoneally injected with 5-FU (150 mg/kg, 200 *μ*L) for 6 days; mice of control group were intraperitoneally injected with PBS (200 *μ*L) for 6 days. All data are presented as mean ± SEM. Statistics: unpaired Student's *t*-test (ns: not significant; ^∗^*p* < 0.05, ^∗∗^*p* < 0.01, ^∗∗∗^*p* < 0.001, and ^∗∗∗∗^*p* < 0.0001).

To determine the optimal conditions for 5-FU-induced damage to BA-MSCs *in vitro*, OP9 cells, a mouse bone marrow stromal cell line, were used in preexperiments. First, OP9 cells were stimulated with 12.5, 25, and 50 *μ*M 5-FU for 24 h and 48 h, and then the apoptosis was detected by flow cytometry. The results showed that the apoptosis of OP9 cells was aggravated as the 5-FU concentration and stimulation time increased (Figure S1a and S1b). Subsequently, BA-MSCs were stimulated with 12.5 *μ*M 5-FU for 24 h and 48 h (Figure 1(a)). As shown in Figure S1c, the number of BA-MSCs slightly decreased after 24 h and was obviously reduced after 48 h. Accordingly, after stimulation with 5-FU for 24 h, the proportion of apoptotic BA-MSCs (annexin V^+^ cells) did not significantly increase compared with that in the control group (Figure S1d). After 5-FU stimulation for 48 h, the proportion of annexin V^+^ BA-MSCs significantly increased, and the number of BA-MSCs dramatically dropped (Figure 1(b)–1(d)). In addition, after treatment with 5-FU for 24 h and 48 h, more BA-MSCs remained in the quiescent phase and pre-DNA synthesis phase (G0/G1), while fewer BA-MSCs entered the DNA synthesis and mitotic phases (S and G2/M), indicating that cell cycle progression of BA-MSCs was significantly inhibited (Figure S1e and Figure 1(e)). These results indicate that *in vitro* treatment with 12.5 *μ*M 5-FU for 48 h could inhibit the proliferation and induce apoptosis of BA-MSCs. In subsequent experiments, we stimulated BA-MSCs with 12.5 *μ*M 5-FU for 48 h to establish a 5-FU-induced BA-MSC injury model *in vitro*.

In *in vivo* experiments, mice were intraperitoneally injected with 5-FU (150 mg/kg) to induce myelosuppression, while the control group was only injected with PBS (Figure 1(f)). After 6 days, routine blood examination was performed to analyze the hematopoiesis in the peripheral circulation. The results showed that the numbers of white blood cells (WBC), RBC, and platelets (PLT) in peripheral blood of the mice injected with 5-FU significantly decreased (Figure S2). To investigate whether BMCs and BA-MSCs in the bone marrow were damaged by 5-FU, BMCs and BA-MSCs were isolated from bone marrow and subjected to the following injury analysis. As shown in Figures 1(g)–1(i), the number of BMCs in the 5-FU group was significantly decreased, and flow cytometry analysis indicated that the proportions of Lin^−^Sca-1^+^c-Kit^+^ cells (HSCs) and Lin^−^Sca-1^−^c-Kit^+^ cells (HPCs) in BMCs were also significantly reduced. These data suggest that 5-FU not only impairs HSPCs but also inhibits hematopoietic function of the bone marrow *in vivo*. Moreover, the number of BA-MSCs decreased, and the number of apoptotic BA-MSCs severely increased (Figures 1(j)–1(l)). In addition, cell cycle analysis showed fewer BA-MSCs in the DNA synthesis and mitotic phases (S and G2/M) (Figure 1(m)). These results illustrate that 5-FU seriously damages both HSPCs and BA-MSCs *in vivo*. Taken together, 5-FU can induce BA-MSC apoptosis and inhibit proliferation of BA-MSCs *in vitro* and *in vivo.*

### 3.2. 5-FU Damages the Hematopoietic Regulatory Function of BA-MSCs *In Vitro* and *In Vivo*

**Figure 2:**
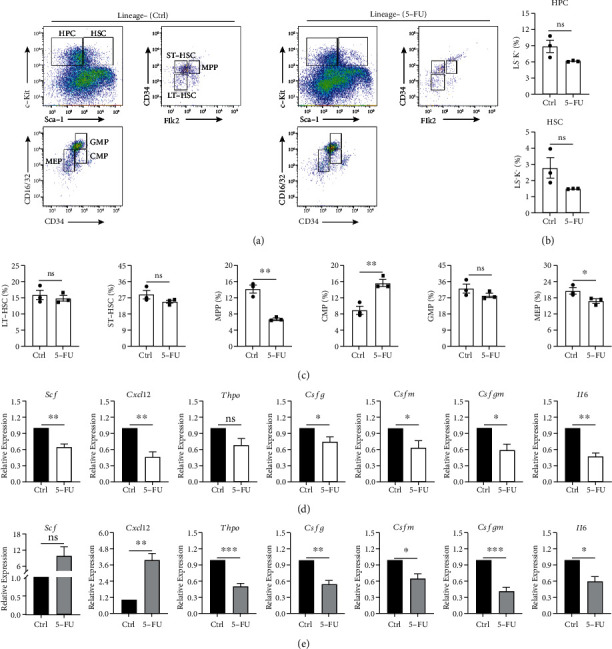
5-FU impairs the hematopoietic regulatory ability of BA-MSCs. (a) Flow cytometric plots of BMCs cocultured with DMSO-treated BA-MSCs and 5-FU-treated BA-MSCs for 2 days. Flow cytometric plots are gated on lineage-negative BMCs and showing cell subsets of HPC (Lin^−^Sca-1^−^c-Kit^+^), HSC (Lin^−^Sca-1^+^c-Kit^+^), LT-HSC (Lin^−^Sca-1^+^c-Kit^+^CD34^low^Flk2^low^), ST-HSC (Lin^−^Sca-1^+^c-Kit^+^CD34^+^Flk2^low^), MPP (Lin^−^Sca-1^+^c-Kit^+^CD34^+^Flk2^+^), CMP (Lin^−^Sca-1^−^c-Kit^+^CD34^+^CD16/32^low^), GMP (Lin^−^Sca-1^−^c-Kit^+^CD34^+^CD16/32^+^), and MEP (Lin^−^Sca-1^−^c-Kit^+^CD34^low^CD16/32^low^) (*n* = 3). (b, c) Flow cytometry analysis of the percentage of HPC, HSC, LT-HSC, ST-HSC, MPP, CMP, GMP, and MEP in the BMCs cocultured with DMSO-treated BA-MSCs and 5-FU-treated BA-MSCs for 2 days (*n* = 3). (d) Gene expressions of *Scf*, *Cxcl12*, *Thpo*, *Csfg*, *Csfm*, *Csfgm*, and *Il6* in DMSO-treated BA-MSCs and 5-FU-treated BA-MSCs were detected by qRT-PCR (*n* = 5). (e) Gene expressions of *Scf*, *Cxcl12*, *Thpo*, *Csfg*, *Csfm*, *Csfgm*, and *Il6* in control mice and 5-FU-induced myelosuppression mice were detected by qRT-PCR (*n* = 5). All data are presented as mean ± SEM. Statistics: unpaired Student's *t*-test (ns: not significant; ^∗^*p* < 0.05, ^∗∗^*p* < 0.01, and ^∗∗∗^*p* < 0.001).

To determine the hematopoietic regulatory function of 5-FU-injured BA-MSCs, freshly isolated BMCs were cocultured with BA-MSCs for 2 days, and the self-renewal and differentiation of HSPCs were detected by flow cytometry. According to Figures 2(a) and 2(b), the frequencies of HSCs and HPCs in the 5-FU group decreased. These results indicate that the ability of BA-MSCs to support HSPC self-renewal was impaired. In addition, the percentages of Lin^−^Sca-1^+^c-Kit^+^CD34^+^Flk2^+^ multipotent progenitors (MPPs) and Lin^−^Sca-1^−^c-Kit^+^CD34^low^CD16/32^low^ megakaryocyte-erythrocyte progenitors (MEPs) significantly decreased, suggesting that the ability of BA-MSCs to support HSPC differentiation was impaired. Only the percentage of Lin^−^Sca-1^−^c-Kit^+^CD34^+^CD16/32^low^ common myeloid progenitors (CMPs) increased (Figure 2(c)). These data indicate that BA-MSCs exhibit poor hematopoietic support and regulatory function after 5-FU-induced injury *in vitro*.

To investigate whether the poor hematopoietic support and regulatory function of BA-MSCs are due to 5-FU effects on hematopoietic regulatory factors, we determined gene expression of stem cell factor (*Scf*), stromal cell-derived factor 1 (*Sdf1*/*Cxcl12*), thrombopoietin (*Thpo*), granulocyte colony-stimulating factor (*Csfg*), macrophage colony-stimulating factor (*Csfm*), granulocyte-macrophage colony-stimulating factor (*Csfgm*), and interleukin-6 (*Il6*) in 5-FU-injured BA-MSCs by qRT-PCR. Interestingly, the mRNA expression of *Scf*, *Cxcl12*, *Thpo*, *Csfg*, *Csfm*, *Csfgm*, and *Il6* was downregulated after stimulation of BA-MSCs with 5-FU *in vitro* (Figure 2(d)). In the 5-FU-induced mouse myelosuppression model, mRNA expression of *Thpo*, *Csfg*, *Csfm*, *Csfgm*, and *Il6* in BA-MSCs was downregulated as well, while that of *Scf* and *Cxcl12* was upregulated, which may be related to the cellular stress response (Figure 2(e)). Altogether, these results indicate that the 5-FU-induced injury impairs the hematopoietic regulatory function of BA-MSCs both *in vitro* and *in vivo.*

### 3.3. 5-FU Impairs the Mitochondrial Dynamics and Function of BA-MSCs

**Figure 3:**
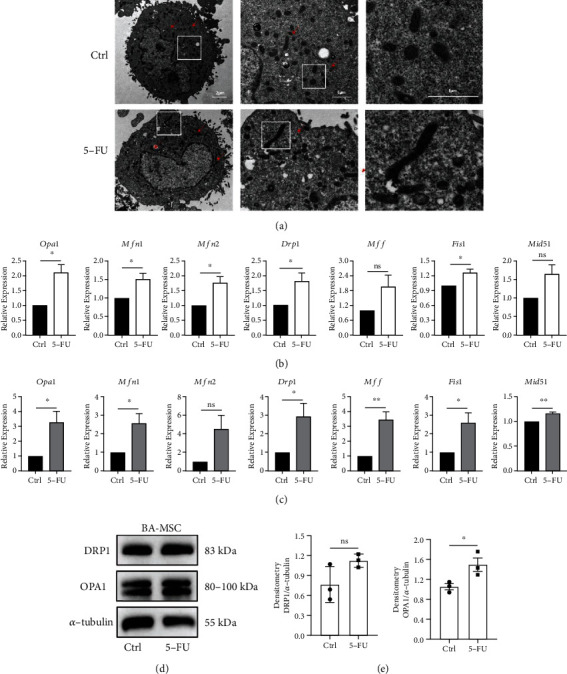
Analysis of mitochondrial dynamics in BA-MSCs after 5-FU chemotherapy. (a) TEM images depicting morphological changes of mitochondria (white rectangles) in control BA-MSCs and 5-FU-injured BA-MSCs. Scale bar, 2 *μ*m, and 1 *μ*m. (b) Gene expressions of *Opa1*, *Mfn1*, *Mfn2*, *Drp1*, *Mff*, *Fis1*, and *Mid51* in DMSO-treated BA-MSCs and 5-FU-treated BA-MSCs were detected by qRT-PCR (*n* = 5). (c) Gene expression of *Opa1*, *Mfn1*, *Mfn2*, *Drp1*, *Mff*, *Fis1*, and *Mid51* in control mice and 5-FU-induced myelosuppression mice were detected by qRT-PCR (*n* = 5). (d) Protein expression of DRP1 and OPA1 in DMSO-treated BA-MSCs and 5-FU-treated BA-MSCs were detected by western blotting. (e) Western blotting analysis of DRP1 and OPA1 in DMSO-treated BA-MSCs and 5-FU-treated BA-MSCs (*n* = 3). All data are presented as mean ± SEM. Statistics: unpaired Student's *t*-test (ns: not significant; ^∗^*p* < 0.05 and ^∗∗^*p* < 0.01).

5-FU is an antimetabolic chemotherapy agent that inhibits cell proliferation. It mainly inhibits thymidylate synthase and thus blocks the formation of thymidine, which is required for DNA synthesis [39]. Since mitochondria are semiautonomous organelles containing DNA, we examined whether 5-FU impairs the mitochondrial dynamics and function in BA-MSCs. First, the morphology of mitochondria in BA-MSCs stimulated with 5-FU was observed by TEM. The results showed that after 5-FU treatment, the number of rod-shaped mitochondria in BA-MSCs increased. In addition, the number of damaged mitochondria in 5-FU-injured BA-MSCs was elevated (Figure 3(a)). Next, we investigated the mRNA expression of mitochondrial fusion genes, such as optic atrophy 1 (*Opa1*), mitofusin 1 *(Mfn1*), and mitofusin 2 (*Mfn2*), and mitochondrial fission genes, such as dynamin-related protein 1 (*Drp1*), mitochondrial fission factor (*Mff*), mitochondrial fission 1 (*Fis1*), and mitochondrial dynamic proteins of 51 kDa (*Mid51*). As shown in Figures 3(b) and 3(c), the mRNA expression of the mitochondrial fusion and fission genes was upregulated *in vitro* and *in vivo* after 5-FU stimulation. We also determined the protein expression levels of DRP1 and OPA1 in 5-FU-damaged BA-MSCs by western blotting. The results of protein band imaging and densitometric analysis showed that the levels of DRP1 and OPA1 in BA-MSCs markedly increased after 5-FU injury, which was consistent with their gene expression levels (Figures 3(d) and 3(e)). These data demonstrate that 5-FU disrupts the balance of the mitochondrial dynamics within BA-MSCs, and the remaining mitochondria responded to 5-FU damage by promoting the fusion and fission between themselves.

**Figure 4:**
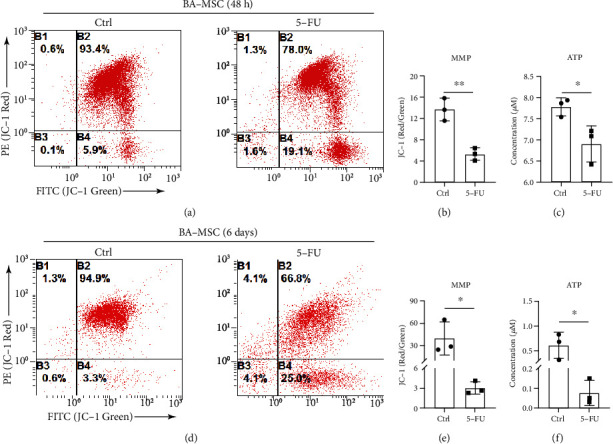
Analysis of mitochondrial function in BA-MSCs after 5-FU chemotherapy. (a) Flow cytometric plots of MMP detection in DMSO-treated BA-MSCs and 5-FU-treated BA-MSCs. (b) Flow cytometry analysis of MMP in DMSO-treated BA-MSCs and 5-FU-treated BA-MSCs (*n* = 3). (c) ATP analysis of DMSO-treated BA-MSCs and 5-FU-treated BA-MSCs (*n* = 3). (d) Flow cytometric plots of MMP detection of BA-MSCs in control mice and 5-FU-induced myelosuppression mice. (e) Flow cytometry analysis of MMP of BA-MSCs in control mice and 5-FU-induced myelosuppression mice (*n* = 3). (f) ATP analysis of BA-MSCs in control mice and 5-FU-induced myelosuppression mice (*n* = 3). All data are presented as mean ± SEM. Statistics: unpaired Student's *t*-test (ns: not significant; ^∗^*p* < 0.05 and ^∗∗^*p* < 0.01).

To further verify the damaging effect of 5-FU on mitochondrial function, the MMP and ATP content were measured in BA-MSCs. The results showed that the MMP and ATP content decreased in BA-MSCs after 5-FU stimulation *in vitro* (Figures 4(a)–4(c)) and *in vivo* (Figures 4(d)–4(f)). Collectively, 5-FU impairs the mitochondrial dynamics and function of BA-MSCs both *in vivo* and *in vitro*.

### 3.4. Transfer of Platelet-Derived Mitochondria to BA-MSCs Attenuates 5-FU-Induced Apoptosis

**Figure 5:**
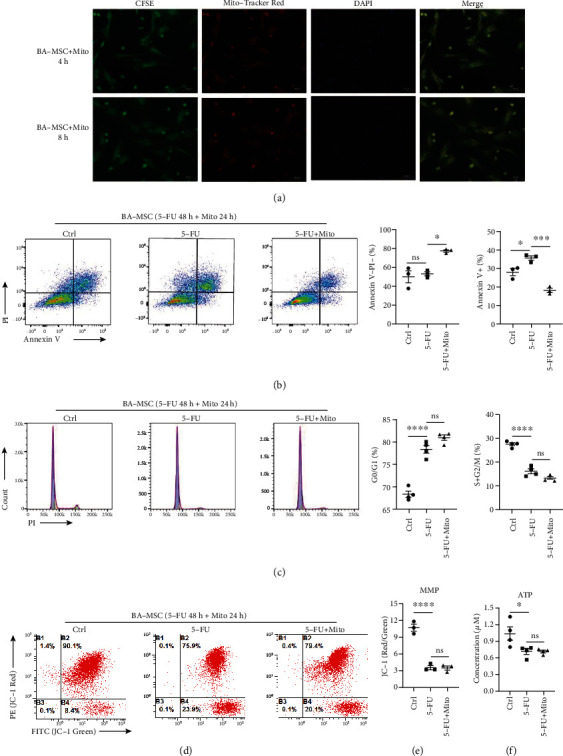
Evaluation of the efficacy of platelet-derived mitochondria in the treatment of BA-MSCs injured by 5-FU. (a) Representative fluorescence images of BA-MSCs after 4 h and 8 h incubation with platelet-derived mitochondria. Mitochondria were previously labeled with MitoTracker Red CMXRos (red signal). The cytoplasm of BA-MSCs was labeled with CFSE (green signal), and the nucleus was labeled with DAPI (blue signal) before detection. (b) After incubation of 5-FU-injured BA-MSCs with platelet-derived mitochondria for 24 h, the apoptosis of BA-MSCs was analyzed by flow cytometry. (c) After incubation of 5-FU-injured BA-MSCs with platelet-derived mitochondria for 24 h, the cell cycle of BA-MSCs was analyzed by flow cytometry. (d, e) After incubation of 5-FU-injured BA-MSCs with platelet-derived mitochondria for 24 h, the MMP of BA-MSCs was analyzed by flow cytometry. (f) After incubation of 5-FU-injured BA-MSCs with platelet-derived mitochondria for 24 h, the ATP of BA-MSCs was analyzed. Control group: BA-MSCs were treated with DMSO for 48 h. 5-FU group: BA-MSCs were treated with 5-FU for 48 h. 5-FU + Mito group: BA-MSCs were treated with 5-FU for 48 h and incubated with platelet-derived mitochondria for 24 h. All data are presented as mean ± SEM. Statistics: one-way ANOVA (ns: not significant; ^∗^*p* < 0.05, ^∗∗∗^*p* < 0.001, and ^∗∗∗∗^*p* < 0.0001).

To investigate whether platelet-derived mitochondria could be transferred to 5-FU-injured BA-MSCs and alleviate the damage of 5-FU on the hematopoietic regulatory function of BA-MSCs, we isolated highly purified platelet-derived mitochondria, which contained the mitochondrial marker proteins TOMM20 and COX IV but did not contain the cytoplasmic proteins GAPDH (Figure S3a). The MMP detection of platelet-derived mitochondria and CCCP-inhibited mitochondria (positive control) indicated that the isolated mitochondria had stable biological function (Figure S3b). As shown in Figure S3c, the isolated mitochondria labeled with MitoTracker Red did not contain platelets and were within the size range of mitochondria (1–2 *μ*m). These results confirm that the isolated high-purity mitochondria have complete structure and normal biological function. To further validate our hypothesis, mitochondria were prelabeled with MitoTracker Red and incubated with BA-MSCs for 8 h. The successful transfer of mitochondria into the cytoplasm of BA-MSCs was observed at 4 h (Figure 5(a)). This result implies that the isolated mitochondria could successfully and efficiently enter the cytoplasm of BA-MSCs.

To investigate the therapeutic effect of mitochondria, BA-MSCs were stimulated with 5-FU for 48 h and then incubated with platelet-derived mitochondria for 24 h, followed by the detection of apoptosis and analysis of the cell cycle of BA-MSCs. Notably, annexin V/PI analysis indicated that the proportion of annexin V^−^PI^−^ viable cells increased, but that of annexin V^+^ apoptotic BA-MSCs decreased after mitochondrial transfer (Figure 5(b)). These data indicate that mitochondria could attenuate 5-FU-induced apoptosis of BA-MSCs. However, the cell cycle progression of BA-MSCs with 5-FU injury did not change after mitochondrial treatment (Figure 5(c)). These results suggest that the damage caused by 5-FU to the self-renewal potential and proliferation ability of BA-MSCs is difficult to be repaired by mitochondrial therapy.

Additionally, we analyzed the MMP and ATP levels in 5-FU-damaged BA-MSCs after mitochondrial treatment. The results showed that the reduced MMP and ATP levels were not corrected by the transfer of platelet-derived mitochondria, indicating that the 5-FU-caused damage to mitochondrial function of BA-MSCs was difficult to repair (Figures 5(d)–5(f)). In conclusion, these results demonstrate that platelet-derived mitochondria could be transferred to BA-MSCs and attenuated the 5-FU-induced apoptosis of BA-MSCs but could not improve the proliferation ability and mitochondrial function of 5-FU-damaged BA-MSCs.

### 3.5. Platelet-Derived Mitochondria Repair the Hematopoietic Regulatory Function of 5-FU-Injured BA-MSCs

**Figure 6:**
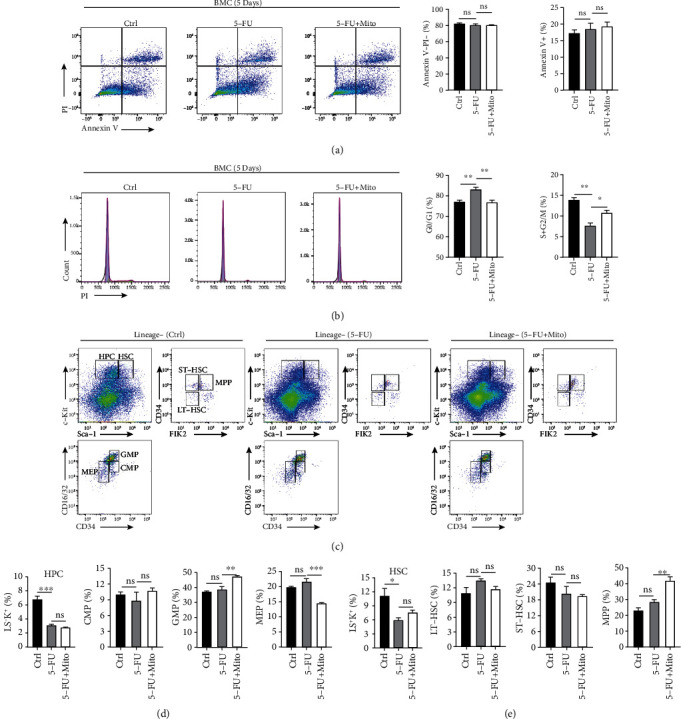
Analysis of hematopoietic regulatory function of BA-MSCs treated with platelet-derived mitochondria after 5-FU chemotherapy. (a) Flow cytometry analysis of apoptosis of BMCs cocultured with BA-MSCs for 5 days. (b) Flow cytometry analysis of cell cycle of BMCs cocultured with BA-MSCs for 5 days. (c) Flow cytometric plots of BMCs cocultured with DMSO-treated BA-MSCs, 5-FU-treated BA-MSCs, and mitochondria-treated 5-FU-damaged BA-MSCs for 2 days. Flow cytometric plots are gated on lineage-negative BMCs and showing cell subsets of HPC, HSC, LT-HSC, ST-HSC, MPP, CMP, GMP, and MEP. (d, e) Flow cytometry analysis of percentage of HPC, HSC, LT-HSC, ST-HSC, MPP, CMP, GMP, and MEP cell subsets (*n* = 3). Control group: BA-MSCs were treated with DMSO for 48 h. 5-FU group: BA-MSCs were treated with 5-FU for 48 h. 5-FU + Mito group: BA-MSCs were treated with 5-FU for 48 h and incubated with platelet-derived mitochondria for 24 h. All data are presented as mean ± SEM. Statistics: one-way ANOVA (ns: not significant; ^∗^*p* < 0.05, ^∗∗^*p* < 0.01, and ^∗∗∗^*p* < 0.001).

Considering that platelet-derived mitochondria could alleviate the apoptosis of 5-FU-damaged BA-MSCs, we next investigated the repair effect of mitochondria on the hematopoietic regulatory function of 5-FU-injured BA-MSCs. BMCs were isolated and cocultured with BA-MSCs (control, 5-FU injury, and mitochondrial treatment groups). After 5 days, the apoptosis and cell cycle phases of BMCs were assessed by flow cytometry. As shown in Figure 6(a), there were no significant differences in the degrees of BMC apoptosis among the control, 5-FU injury, and mitochondrial treatment groups. However, the cell cycle of BMCs in the 5-FU injury group was arrested, unlike that in the control group. In particular, more cells entered the G0/G1 phases, while the proportion of cells in the S and G2/M phases decreased, indicating that the proliferation of BMCs was inhibited in the 5-FU injury group. Interestingly, compared with those in the 5-FU injury group, the proportion of BMCs in the G0/G1 phases significantly decreased, and that in the S and G2/M phases significantly increased in the mitochondrial treatment group, in which the proportion of BMCs in each cell cycle phase was closer to that in the control group (Figure 6(b)). These results suggest that 5-FU impairs the ability of BA-MSCs to regulate the cell cycle of BMCs and support their proliferation. Meanwhile, mitochondria could alleviate the 5-FU-caused damage to the BA-MSCs' ability to regulate the cell cycle of BMCs.

To further evaluate whether the hematopoietic regulatory function of 5-FU-injured BA-MSCs could be repaired by mitochondria, freshly isolated BMCs were cocultured with BA-MSCs (control, 5-FU injury, and mitochondrial treatment groups), as described above. After 2 days, the self-renewal and differentiation of HSPCs were assessed by flow cytometry (Figure 6(c)). The data showed that there were no significant differences in the frequencies of HSCs and HPCs between the 5-FU injury and mitochondrial treatment groups (Figures 6(d) and 6(e)). Analysis of the proportions of LT-HSCs, ST-HSCs, and CMPs between the 5-FU injury and mitochondrial treatment groups also showed no significant differences. These data suggest that mitochondria could not repair the ability of 5-FU-damaged BA-MSCs to support the self-renewal of HSPCs. The percentages of MPPs and GMPs increased, but that of MEPs decreased in the mitochondrial treatment group compared with those in the control and 5-FU injury groups, indicating that mitochondria might only slightly affect the terminal differentiation of HSPCs (Figures 6(d) and 6(e)). These results demonstrate that mitochondria could slightly improve the ability of 5-FU-damaged BA-MSCs to support the terminal differentiation of HSPCs but not HSPC self-renewal and early differentiation. In conclusion, mitochondria can alleviate the 5-FU-caused damage to the BA-MSC ability to regulate the cell cycle of BMCs; however, it is difficult to completely alleviate the damage to the hematopoietic regulatory function of BA-MSCs by mitochondrial transfer.

### 3.6. Mitochondrial Transfer Increases the Expression of Hematopoietic Regulatory Factor Genes and Regulates Mitochondrial Dynamics in BA-MSCs with 5-FU Injury

**Figure 7:**
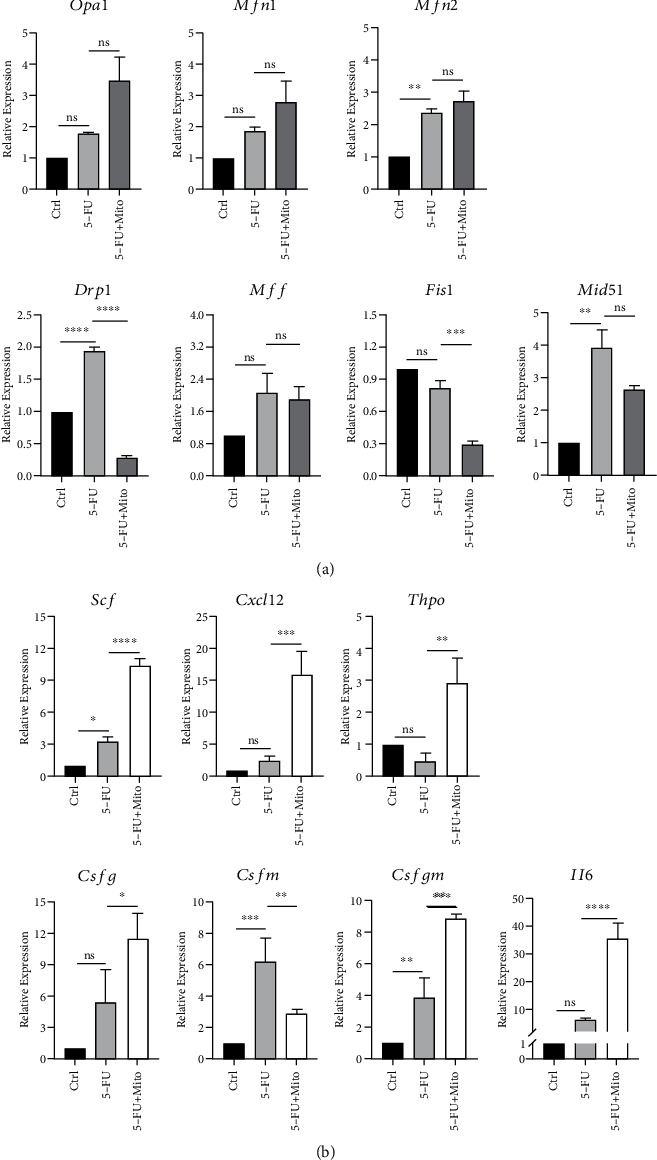
Analysis of mRNA expression of hematopoietic regulatory factor genes and mitochondrial fusion/fission genes in BA-MSCs after mitochondrial treatment of BA-MSCs damaged by 5-FU. (a) Gene expressions of *Opa1*, *Mfn1*, *Mfn2*, *Drp1*, *Mff*, *Fis1*, and *Mid51* in DMSO-treated BA-MSCs, 5-FU-treated BA-MSCs, and mitochondria-treated 5-FU-damaged BA-MSCs were detected by qRT-PCR (*n* = 3). (b) Gene expressions of *Scf*, *Cxcl12*, *Thpo*, *Csfg*, *Csfm*, *Csfgm*, and *Il6* in DMSO-treated BA-MSCs, 5-FU-treated BA-MSCs, and mitochondria-treated 5-FU-damaged BA-MSCs were detected by qRT-PCR (*n* = 3). All data are presented as mean ± SEM. Statistics: one-way ANOVA (ns: not significant; ^∗^*p* < 0.05, ^∗∗^*p* < 0.01, ^∗∗∗^*p* < 0.001, and ^∗∗∗∗^*p* < 0.0001).

**Figure 8:**
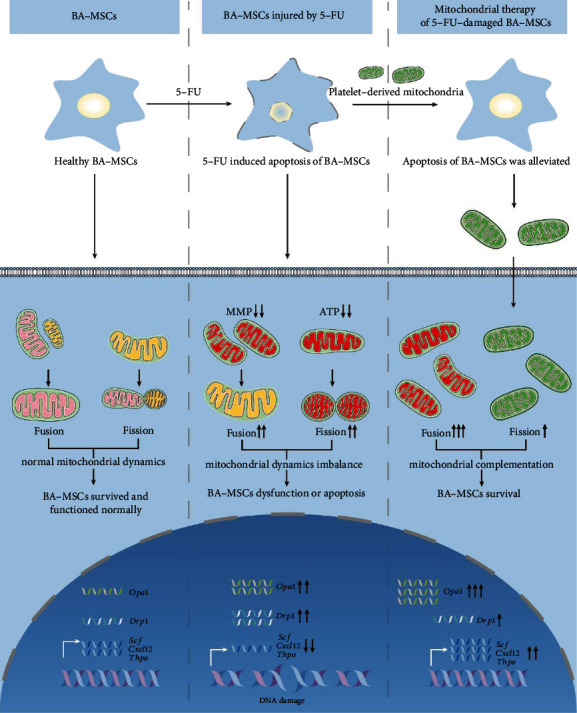
Schematic summary of the process and mechanism of our study. 5-FU induces the apoptosis of BA-MSCs, and platelet-derived mitochondria attenuates 5-FU-induced apoptosis of BA-MSCs. 5-FU chemotherapy can damage mitochondrial dynamics, mitochondrial functions, and expression of hematopoietic regulatory factor genes in BA-MSCs. However, under the treatment of platelet-derived mitochondria, the damage of mitochondria and hematopoietic regulatory function of BA-MSCs was alleviated. Mitochondria marked in pink: healthy mitochondria of BA-MSCs. Mitochondria marked in yellow: slightly damaged mitochondria of BA-MSCs. Mitochondria marked in red: severely damaged mitochondria of BA-MSCs. Mitochondria marked in green: platelet-derived healthy mitochondria.

As we have demonstrated above, platelet-derived mitochondria can transfer to BA-MSCs to reduce the apoptosis and partially improve the hematopoietic regulatory ability of 5-FU-injured BA-MSCs. Since apoptosis is associated with the mitochondrial dynamics of cell, and the hematopoietic regulatory function is closely related to the level of hematopoietic regulatory factors, we further explored the gene expression of mitochondrial fusion and fission genes and hematopoietic regulatory factor genes in 5-FU-injured BA-MSCs after mitochondrial therapy. As shown in Figure 7(a), the mRNA expression of mitochondrial fusion genes (*Opa1*, *Mfn1*, and *Mfn2*) in mitochondria treatment group was higher than that in the 5-FU injury group and control group. On the contrary, the mRNA expression of mitochondrial fission genes (*Drp1*, *Fis1*, and *Mid51*) in mitochondria treatment group was lower than that in the 5-FU injury group. These data indicate that mitochondrial fusion increased and mitochondrial fission decreased after mitochondria treatment in 5-FU-injured BA-MSCs. This suggests that platelet-derived healthy mitochondria can help relieve cell stress and 5-FU-induced apoptosis by increasing fusion with damaged mitochondria in 5-FU-injured BA-MSCs. In addition, when 5-FU stimulation was removed for 24 h, the expression of hematopoietic regulatory factor genes (*Scf*, *Cxcl12*, *Csfg*, *Csfm*, *Csfgm*, and *Il6*) in BA-MSCs in the 5-FU injury group was upregulated, but the expression of hematopoietic regulatory factor genes (*Scf*, *Cxcl12*, *Thpo*, *Csfg*, *Csfgm*, and *Il6*) in the mitochondrial treatment group was much more upregulated than that in the 5-FU injury group (Figure 7(b)). These findings suggest that platelet-derived mitochondria may improve the hematopoietic regulatory ability of 5-FU-injured BA-MSCs by increasing the expression of hematopoietic regulatory factor genes. Overall, these results demonstrate that platelet-derived mitochondria alleviate apoptosis of 5-FU-injured BA-MSCs by regulating mitochondrial dynamics and improve hematopoietic regulatory ability of 5-FU-injured BA-MSCs by upregulating the expression of hematopoietic regulatory factor genes (Figure 8).

## 4. Discussion

In the current study, we found that 5-FU induced the apoptosis and blocked cell cycle progression of BA-MSCs both *in vitro* and *in vivo*. The number of BA-MSCs was significantly reduced upon stimulation with 5-FU *in vitro* or in myelosuppression mice, and the proliferation of BA-MSCs was also inhibited, which had a great negative effect on hematopoiesis. Regarding the function of BA-MSCs, 5-FU impaired the ability of BA-MSCs to regulate hematopoiesis. In addition, the balance of the mitochondrial dynamics in BA-MSCs was adversely affected by 5-FU, and mitochondrial function was seriously damaged. Mitochondria are important organelles for energy supply and substance metabolism in eukaryocytes. The severe damage to mitochondria is one of the reasons for the serious injury caused by 5-FU to BA-MSCs. Surprisingly, treatment with platelet-derived mitochondria reduced the 5-FU-induced apoptosis and partially restored the impaired hematopoietic regulatory function of BA-MSCs (Figure 8). However, platelet-derived mitochondria could not repair the 5-FU-induced damage to the MMP and restore the ATP level in BA-MSCs, which suggested that the therapeutic effect of platelet-derived mitochondria on 5-FU-injured BA-MSCs was limited. These results indicate that 5-FU causes severe damage to BA-MSCs, which could not be repaired by mitochondrial transfer alone. Our findings affirmed the importance of recognizing the damage caused by chemotherapy to BA-MSCs. The transplantation of mitochondria into injured BA-MSCs for treatment is an innovation of this study. This work provides a research basis and a direction for the treatment of chemotherapy-injured BA-MSCs.

In this study, 5-FU-injured BA-MSCs showed much more mitochondrial fusion and fission both in genes and protein expression (Figures 3(b)–3(e)). These results indicate that 5-FU stimulation leads to changes in mitochondrial dynamics. The reason for such changes may be that the cells, in order to defend against stress, thereby increasing mitochondrial fusion to share material and energy and increasing mitochondrial fission to clear the damaged and nonfunctional mitochondria. Damaged mitochondria were discarded through mitochondrial fission to maintain cellular homeostasis. This is helpful for BA-MSCs to alleviate 5-FU-induced injury (Figure 8). When mitochondria fail to respond to harmful 5-FU stimuli by autonomic regulation, cells become dysfunctional and may even die. Since mitochondrial function and dynamics are closely related to the apoptosis and stem cell potential of BA-MSCs [40, 41], we proposed a hypothesis that transplantation of platelet-derived mitochondria into BA-MSCs could repair the mitochondrial damage caused by 5-FU. Interestingly, our results demonstrate that platelet-derived mitochondria could be transferred to 5-FU-injured BA-MSCs and alleviate the damaging effects of 5-FU on the hematopoietic regulatory function of BA-MSCs by repairing the injury of mitochondria. These results demonstrate the importance of repairing mitochondrial damage in BA-MSCs and the feasibility of using platelet-derived mitochondria to treat the damage of BA-MSCs caused by chemotherapy.

Platelets are widely used in the clinic [42, 43]. In addition to regulating hemostasis in blood vessels [30], platelets play an important role in innate immunity [44, 45], tumor growth [46–48], and vascular extravasation [49–51]. With the discovery of platelet function, platelet derivatives have been proposed as alternatives to FBS for *in vitro* expansion of MSCs to limit the risk of zoonotic diseases and xenogeneic immune reactions in transplanted hosts [31]. Platelet lysate (PL), which contains platelet-derived growth factors, can promote the expansion of MSCs *in vitro*. PL is obtained from the platelet-rich plasma (PRP) by repeated freezing and thawing. Studies have shown that PL-expanded MSCs have a faster proliferation rate than FBS-expanded MSCs, which has been applied in clinical practice [31]. Other studies have suggested that the PRP combined with MSCs can improve the efficacy of cell therapy for tissue repair [32, 52–55]. In addition, PRP treatment was shown to stimulate the proangiogenic potential of MSCs *in vitro* by increasing their secretion of soluble factors, such as VEGF and SDF-1 [32, 56, 57]. Moreover, PRP treatment improved the survival and activated the proliferation of MSCs *in vitro* and *in vivo* [32, 58–62], and these effects were accompanied by changes in MSC energy metabolism, including the oxygen consumption rate and mitochondrial ATP production [32]. However, the exact mechanism underlying the PRP effects remains unclear.

Levoux et al. found that platelets could promote metabolic reprogramming of MSCs through mitochondrial transfer, thereby activating the fatty acid synthesis pathway in MSCs [33]. Therefore, pretreatment with platelets can increase the secretion of proangiogenic factors and promote the wound-healing ability of MSCs. These results reveal a novel mechanism by which platelets enhance the properties of MSCs and confirm the potential of transplantation of platelet-derived mitochondria to improve the capacity of MSCs as stem cell therapeutics [32, 33, 63]. A low cell viability and poor engraftment are obvious limitations of stem cell therapy [64, 65]. A recent study has reported that the transfer of exogenous mitochondria to ADSCs can effectively increase their energy levels for efficient engraftment. Mitochondrial transfer leads to a dramatic enhancement of the bioenergetics, which further leads to changes in the secretome characteristics of ADSCs. The increased production of ATP and increased expression of cyclin-dependent kinases 1 and 2 may contribute to enhanced proliferation, migration, and differentiation abilities of ADSCs *in vitro* [29]. Taken together, mitochondrial transplantation is a promising approach to engineering stem cells for tissue regeneration [66, 67]. Platelets are anucleate cell-like structures with mitochondria and are abundant in the blood [30, 68]. They are a convenient and low-cost source of mitochondria. Through simple venipuncture, platelets can easily be obtained from peripheral blood with little damage to the body [69, 70]. Therefore, isolation of platelet-derived mitochondria is minimally invasive and easy to perform. In our future research, we will explore wider applications and in-depth mechanisms of transplantation of platelet-derived mitochondria, especially for the repair of chemotherapy-caused BA-MSC damage. Further understanding of the damage caused by chemotherapy to BA-MSCs, especially to mitochondria, and of the effect of chemotherapy on mitochondrial fusion and fission is of great significance. Our study demonstrated that the transplantation of platelet-derived mitochondria could repair the mitochondrial damage in BA-MSCs and promote their hematopoietic regulatory function after chemotherapy, which has a broad clinical application prospect. These findings can further improve the clinical application potential of platelets and BA-MSCs and provide a new direction for improving the efficiency of HSC transplantation and hematopoietic recovery after chemotherapy.

Although we made some progress in the study of the 5-FU-caused damage to BA-MSC function and mitochondrial dynamics and demonstrated that platelet-derived mitochondria could alleviate the apoptosis and improve the hematopoietic regulatory ability of 5-FU-injured BA-MSCs, there are still some limitations to our study. First, we did not reveal the relationship between the mitochondrial dynamics and the multilineage (osteogenic, chondrogenic, and adipogenic) differentiation potential of BA-MSCs after 5-FU injury. Second, we found that platelet-derived mitochondria could be transferred to BA-MSCs, but the MMP and ATP level of 5-FU-injured BA-MSCs were not improved after treatment with mitochondria, and the reason for this phenomenon was not investigated. Third, we did not thoroughly elucidate the mechanism of 5-FU damage to BA-MSC mitochondria nor did we investigate the signaling pathways and metabolic changes in platelet-derived mitochondria involved in the repair of 5-FU-induced damage to BA-MSCs. Finally, no effective treatment was found to repair the 5-FU-induced BA-MSC damage. Thus, further investigation is needed to clarify whether incubation of mouse platelet-derived mitochondria or platelets with BA-MSCs can improve the efficacy of mitochondria in the treatment of 5-FU-injured BA-MSCs. Despite these limitations, further research to solve the above problems and deficiencies will deepen our knowledge of the mechanism of chemotherapy-induced mitochondrial damage in BA-MSCs and the application prospect for platelet-derived mitochondria in the treatment of chemotherapy-induced damage to BA-MSCs. In-depth research on related issues will help find methods to alleviate the damage to BA-MSCs during chemotherapy and clarify its underlying mechanism. The treatment of chemotherapy-induced BA-MSC injury is of great significance for hematopoietic recovery after chemotherapy.

## 5. Conclusions

In conclusion, our findings revealed the damage of the classical chemotherapy drug 5-FU to BA-MSCs and mitochondria *in vitro* and *in vivo*, as well as the therapeutic effect of transferring platelet-derived mitochondria to 5-FU-injured BA-MSCs. Our study highlights the severity of the BA-MSC injury caused by chemotherapy drugs and the importance of repairing BA-MSC injury for hematopoietic recovery. This study lays a foundation for the research of chemotherapy-related damage and repair of BA-MSC mitochondria and deepens our understanding of mitochondrial dynamics changes in BA-MSCs due to chemotherapy-related damage.

## Data Availability

The data used and/or analyzed during the current study are available from the corresponding authors on reasonable request.

## Abbreviations

ADSCs: Adipose-derived mesenchymal stem cells
5-FU: 5-Fluorouracil
MSCs: Mesenchymal stem/stromal cells
BA-MSCs: Bone-associated mesenchymal stem cells
GVHD: Graft-vs-host-disease
HSPCs: Hematopoietic stem/progenitor cells
HSCs: Hematopoietic stem cells
HPCs: Hematopoietic progenitor cells
Mito: Mitochondria
WBC: White blood cells
RBC: Red blood cells
PLT: Platelets
MMP: Mitochondrial membrane potential
ATP: Adenosine triphosphate
LT-HSCs: Long-term hematopoietic stem cells
ST-HSCs: Short-term hematopoietic stem cells
MPPs: Multipotent progenitors
CMPs: Common myeloid progenitors
GMPs: Granulocyte-macrophage progenitors
MEPs: Megakaryocyte-erythrocyte progenitors
*Scf*: Stem cell factor
*Sdf1/Cxcl12*: Stromal cell-derived factor 1
*Thpo*: Thrombopoietin
*Csfg*: Granulocyte colony-stimulating factor
*Csfm*: Macrophage colony-stimulating factor
*Csfgm*: Granulocyte-macrophage colony-stimulating factor
*Il6*: Interleukin-6
*Opa1*: Optic atrophy 1
*Mfn1*: Mitofusin 1
*Mfn2*: Mitofusin 2
*Drp1*: Dynamin-related protein 1
*Mff*: Mitochondrial fission factor
*Fis1*: Mitochondrial fission 1
*Mid51*: Mitochondrial dynamic proteins of 51 kDa
PBS: Phosphate buffer saline
qRT-PCR: Quantitative real-time PCR
RLU: Luminescence
FBS: Fetal bovine serum
PL: Platelet lysate
PRP: Platelet-rich plasma
DMSO: Dimethyl sulfoxide
CFSE: Carboxyfluorescein diacetate succinimidyl ester.

## Supplementary Materials

Supplementary Materials Supplementary Figure S1: analysis of time and concentration of 5-FU in incubation with BA-MSCs to induce apoptosis *in vitro*. Supplementary Figure S2: changes of WBC, RBC, and PLT in peripheral blood of 5-FU-induced myelosuppression mice. Supplementary Figure S3: identification of platelet-derived mitochondria and evaluation of therapeutic efficacy in the treatment of 5-FU-injured BA-MSCs. Supplementary Figure S4: schematic of platelet-derived mitochondria to treat BA-MSCs with 5-FU injury. Supplementary Table S1: primer sequences in quantitative real-time PCR.
