# Supplementary material for: Platelet-Derived Mitochondria Attenuate 5-FU-Induced Injury to Bone-Associated Mesenchymal Stem Cells
Source: Stem Cells Int. 2023 Jan 30;2023:7482546. doi: 10.1155/2023/7482546 (PMC9902133; doi:10.1155/2023/7482546)
Supplement: Supplementary Materials — Supplementary Figure S1: analysis of time and concentration of 5-FU in incubation with BA-MSCs to induce apoptosis in vitro. Supplementary Figure S2: changes of WBC, RBC, and PLT in peripheral blood of 5-FU-induced myelosuppression mice. Supplementary Figure S3: identification of platelet-derived mitochondria and evaluation of therapeutic efficacy in the treatment of 5-FU-injured BA-MSCs. Supplementary Figure S4: schematic of platelet-derived mitochondria to treat BA-MSCs with 5-FU injury. Supplementary Table S1: primer sequences in quantitative real-time PCR. [file 7482546.f1.docx]

**Supplementary Material**

**The following supplementary tables and figures are presented:**

Supplementary Figure S1: Analysis of time and concentration of 5-FU in incubation with BA-MSCs to induce apoptosis *in vitro*.

Supplementary Figure S2: Changes of WBC, RBC and PLT in peripheral blood of 5-FU-induced myelosuppression mice.

Supplementary Figure S3: Identification of platelet-derived mitochondria and evaluation of therapeutic efficacy in the treatment of 5-FU-injured BA-MSCs.

Supplementary Figure S4: Schematic of platelet-derived mitochondria to treat BA-MSCs with 5-FU injury.

Supplementary Table S1: Primer sequences in quantitative real-time PCR.


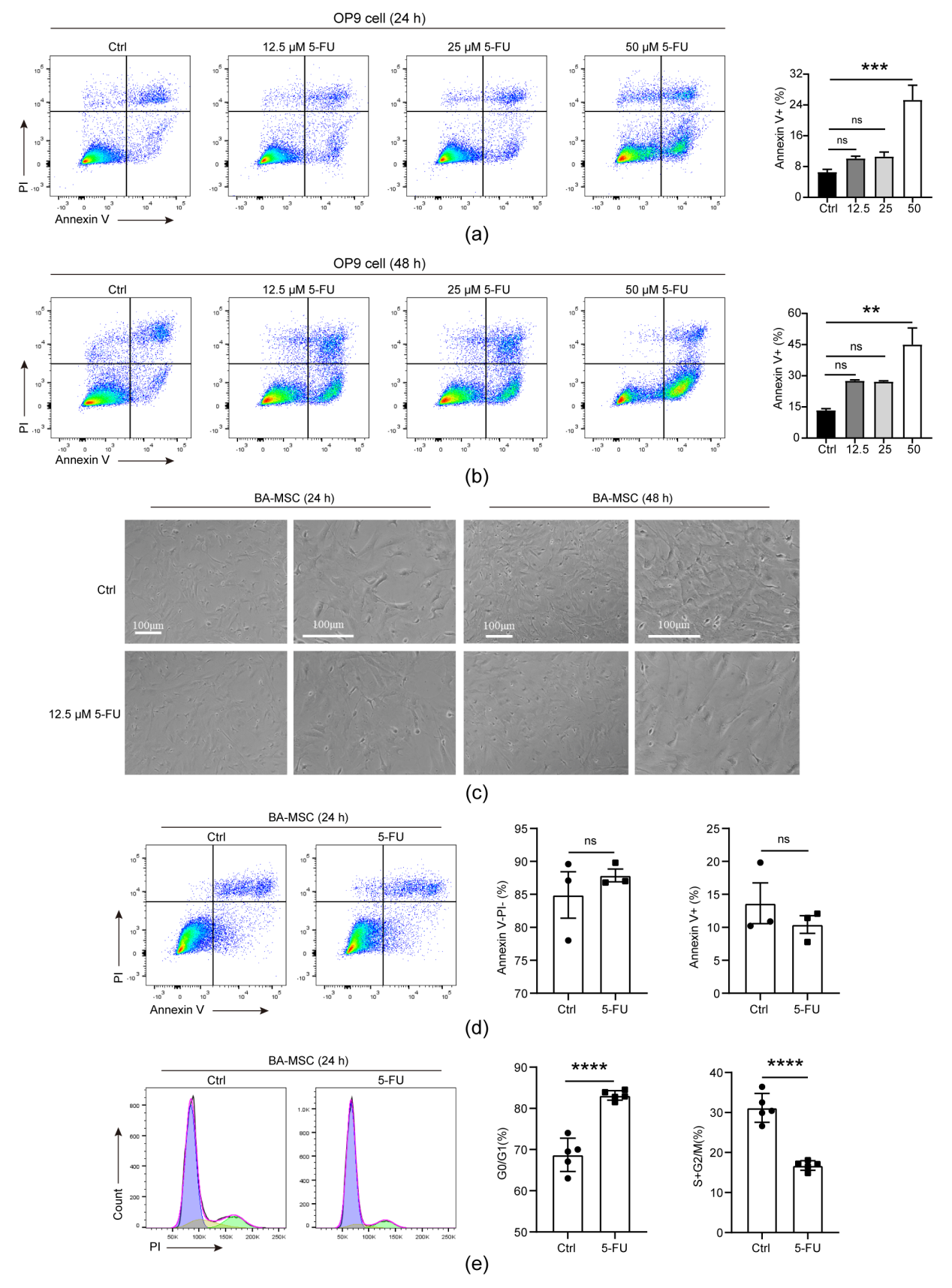


Figure S1: Analysis of time and concentration of 5-FU in incubation with BA-MSCs to induce apoptosis *in vitro*. (a) Flow cytometry analysis of apoptosis of OP9 cells incubated with 12.5 μM, 25 μM and 50 μM 5-FU for 24 h (n=3). (b) Flow cytometry analysis of apoptosis of OP9 cells incubated with 12.5 μM, 25 μM and 50 μM 5-FU for 48 h (n=3). (c) The morphology of BA-MSCs stimulated with 12.5 μM 5-FU for 24 h and 48 h was observed under microscope. (d) Flow cytometry analysis of apoptosis of BA-MSCs incubated with 12.5 μM 5-FU for 24 h (n=3). (e) Flow cytometry analysis of cell cycle of BA-MSCs incubated with 12.5 μM 5-FU for 24 h (n=5). All data are presented as mean ± SEM. Statistics: one-way ANOVA and unpaired Student’s t-test (ns: not significant; ***p* < 0.01, ****p* < 0.001, *****p* < 0.0001).


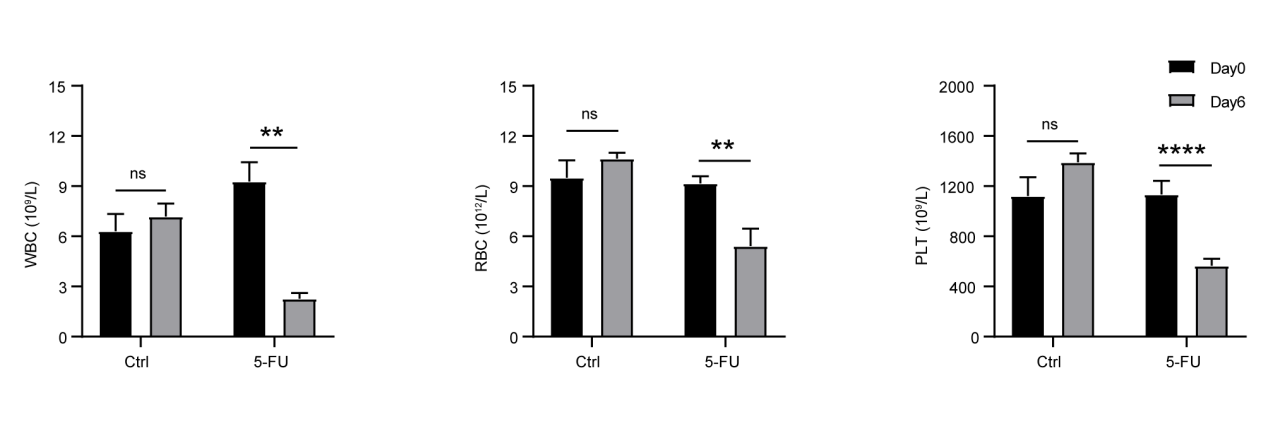


Figure S2: Changes of WBC, RBC and PLT in peripheral blood of 5-FU-induced myelosuppression mice. Blood routine analysis of WBC, RBC and PLT in myelosuppression mice (n=4). All data are presented as mean ± SEM. Statistics: unpaired Student’s t-test (ns: not significant; ***p* < 0.01, *****p* < 0.0001).


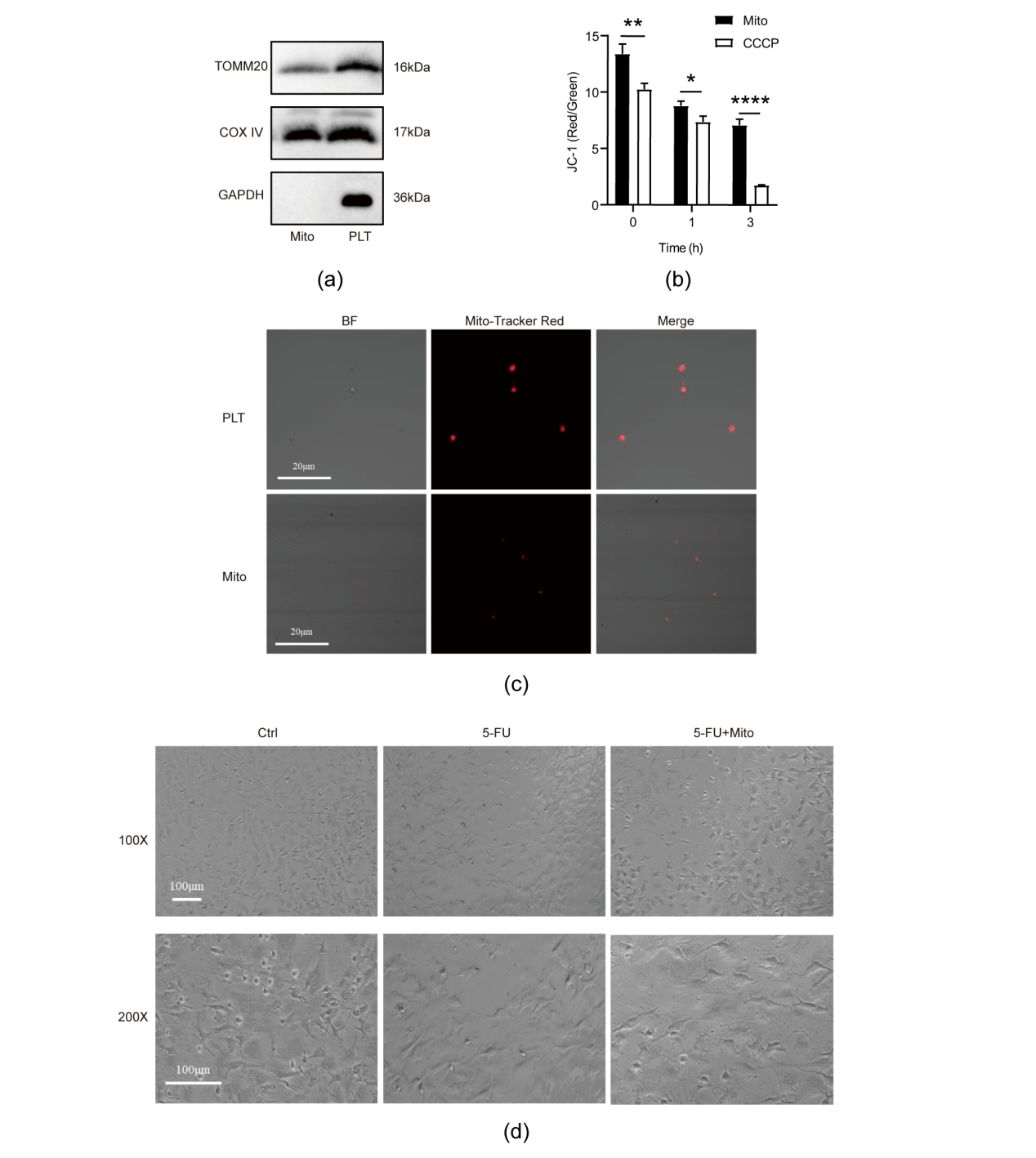


Figure S3: Identification of platelet-derived mitochondria and evaluation of therapeutic efficacy in the treatment of 5-FU-injured BA-MSCs. (a) Western blotting analysis of the expression of mitochondrial proteins TOMM20 and COX Ⅳ in extracted mitochondria and platelets. (b) MMP analysis of extracted platelet-derived mitochondria and mitochondria treated with CCCP (n=3). Carbonyl Cyanide 3-chlorophenylHydraZone (CCCP) is a potent mitochondrial oxidative phosphate de-coupling agent, causing a loss of membrane potential on both sides of the inner mitochondrial membrane. (c) Representative confocal microscopy pictures of platelets and extracted mitochondria. Mitochondria were labeled with MitoTracker Red CMXRos. (d) The morphology analysis of BA-MSCs stimulated with 12.5 μM 5-FU for 48 h and then treated with platelet-derived mitochondria for 24 h. All data are presented as mean ± SEM. Statistics: unpaired Student’s t-test (ns: not significant; **p* < 0.05, ***p* < 0.01, *****p* < 0.0001).


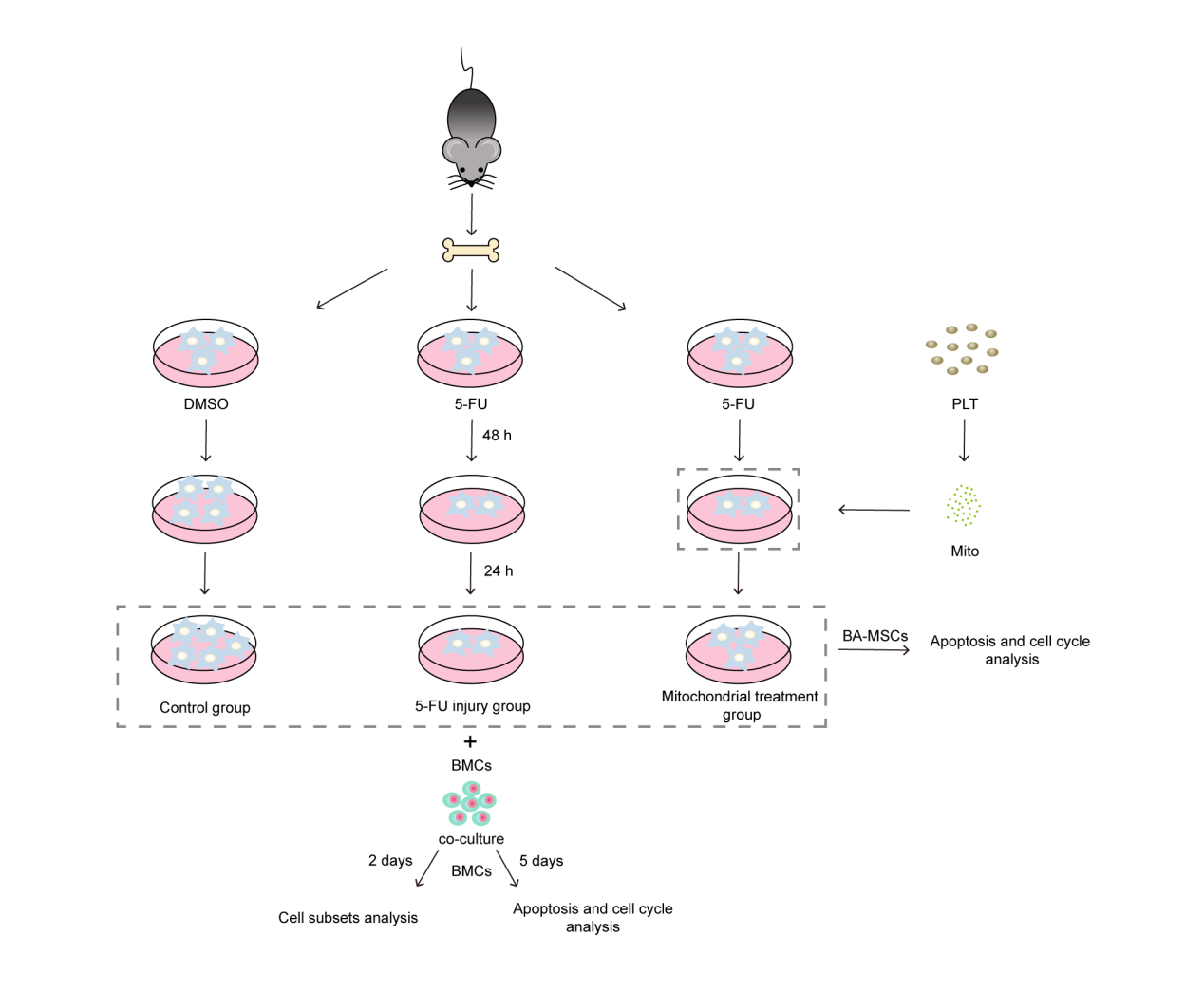


Figure S4: Schematic of platelet-derived mitochondria to treat BA-MSCs with 5-FU injury. Firstly, BA-MSCs were stimulated with 5-FU for 48 h to induce apoptosis of BA-MSCs, and then platelet-derived mitochondria were used to treat BA-MSCs with 5-FU injury for 24 h. Finally, the apoptosis and hematopoietic regulatory ability of BA-MSCs were detected after mitochondrial treatment of 5-FU-damaged BA-MSCs.

| Gene | Primer-F (5’-3’) | Primer-R (5’-3’) |
| --- | --- | --- |
| Scf | GAATCTCCGAAGAGGCCAGAA | GCTGCAACAGGGGGTAACAT |
| Cxcl12 | TGCATCAGTGACGGTAAACCA | TTCTTCAGCCGTGCAACAATC |
| Csfg | ATGGCTCAACTTTCTGCCCAG | CTGACAGTGACCAGGGGAAC |
| Csfm | GGCTTGGCTTGGGATGATTCT | GAGGGTCTGGCAGGTACTC |
| Csfgm | GGCCTTGGAAGCATGTAGAGG | GGAGAACTCGTTAGAGACGACTT |
| Il6 | TAGTCCTTCCTACCCCAATTTCC | TTGGTCCTTAGCCACTCCTTC |
| Thpo | GGCCATGCTTCTTGCAGTG | AGTCGGCTGTGAAGGAGGT |
| Opa1 | TGGAAAATGGTTCGAGAGTCAG | CATTCCGTCTCTAGGTTAAAGCG |
| Mfn1 | CCTACTGCTCCTTCTAACCCA | AGGGACGCCAATCCTGTGA |
| Mfn2 | ACCCCGTTACCACAGAAGAAC | AAAGCCACTTTCATGTGCCTC |
| Drp1 | TTACGGTTCCCTAAACTTCACG | GTCACGGGCAACCTTTTACGA |
| Mid51 | GGTGAGCGCAAAGGGAAGAA | AATGCCCAACATAGCTGCTCC |
| Fis1 | TGTCCAAGAGCACGCAATTTG | CCTCGCACATACTTTAGAGCCTT |
| Mff | AGCTGCCGCCACTTCTAATC | TGCATCTACCACAGTCATGTCA |
| Gapdh | AGGTCGGTGTGAACGGATTTG | TGTAGACCATGTAGTTGAGGTCA |

Table S1: Primer sequences in quantitative real-time PCR.
